# A novel *TcS* allele conferring the high-theacrine and low-caffeine traits and having potential use in tea plant breeding

**DOI:** 10.1093/hr/uhac191

**Published:** 2022-08-25

**Authors:** Hong Zhong, Yi Wang, Fu-Rong Qu, Meng-Yuan Wei, Chen-Yu Zhang, Hao-Ran Liu, Liang Chen, Ming-Zhe Yao, Ji-Qiang Jin

**Affiliations:** Key Laboratory of Biology, Genetics and Breeding of Special Economic Animals and Plants, Ministry of Agriculture and Rural Affairs; Tea Research Institute of the Chinese Academy of Agricultural Sciences, Hangzhou 310008, China; Key Laboratory of Biology, Genetics and Breeding of Special Economic Animals and Plants, Ministry of Agriculture and Rural Affairs; Tea Research Institute of the Chinese Academy of Agricultural Sciences, Hangzhou 310008, China; Key Laboratory of Biology, Genetics and Breeding of Special Economic Animals and Plants, Ministry of Agriculture and Rural Affairs; Tea Research Institute of the Chinese Academy of Agricultural Sciences, Hangzhou 310008, China; Key Laboratory of Biology, Genetics and Breeding of Special Economic Animals and Plants, Ministry of Agriculture and Rural Affairs; Tea Research Institute of the Chinese Academy of Agricultural Sciences, Hangzhou 310008, China; Key Laboratory of Biology, Genetics and Breeding of Special Economic Animals and Plants, Ministry of Agriculture and Rural Affairs; Tea Research Institute of the Chinese Academy of Agricultural Sciences, Hangzhou 310008, China; Key Laboratory of Biology, Genetics and Breeding of Special Economic Animals and Plants, Ministry of Agriculture and Rural Affairs; Tea Research Institute of the Chinese Academy of Agricultural Sciences, Hangzhou 310008, China; Key Laboratory of Biology, Genetics and Breeding of Special Economic Animals and Plants, Ministry of Agriculture and Rural Affairs; Tea Research Institute of the Chinese Academy of Agricultural Sciences, Hangzhou 310008, China

## Abstract

Theacrine (1,3,7,9-tetramethyluric acid) is a natural product with remarkable pharmacological activities such as antidepressant, sedative and hypnotic activities, while caffeine (1,3,7-trimethylxanthine) has certain side effects to special populations. Hence, breeding tea plants with high theacrine and low caffeine will increase tea health benefits and promote consumption. In this study, we construct an F_1_ population by crossing ‘Zhongcha 302’ (theacrine-free) and a tea germplasm ‘Ruyuan Kucha’ (RY, theacrine-rich) to identify the causal gene for accumulating theacrine. The results showed that the content of theacrine was highly negatively correlated with caffeine (R^2^ > 0.9). Bulked segregant RNA sequencing analysis, molecular markers and gene expression analysis indicated that the theacrine synthase (TcS) gene was the candidate gene. The *TcS* was located in the nucleus and cytoplasm, and the theacrine can be detected in stably genetic transformed tobacco by feeding the substrate 1,3,7-trimethyluric acid. Moreover, an *in vitro* enzyme activity experiment revealed that the 241st amino acid residue was the key residue. Besides, we amplified the promoter region in several tea accessions with varied theacrine levels, and found a 234-bp deletion and a 271-bp insertion in RY. Both GUS histochemical analysis and dual-luciferase assay showed that *TcS* promoter activity in RY was relatively high. Lastly, we developed a molecular marker that is co-segregate with high-theacrine individuals in RY’s offspring. These results demonstrate that the novel *TcS* allele in RY results in the high-theacrine and low-caffeine traits and the developed functional marker will facilitate the breeding of characteristic tea plants.

## Introduction

Purine alkaloids, as common secondary metabolites of purine nucleotides in the plant kingdom, consist of the purine ring as the basic skeleton and form different purine bases through oxidation and methylation. The typical purine alkaloids in tea plants include caffeine (1,3,7-trimethylxanthine), theobromine (3,7-dimethylxanthine), and theacrine (1,3,7,9-tetramethyluric acid), etc [1]. Our previous study found that more than 93% of the 403 tea accessions contained 25.0–45.0 mg·g^−1^ of caffeine, while the average content of theobromine was 2.4–4.3 mg·g^−1^ and theacrine only accumulated in a few tea accessions, indicating that most tea plants were rich in caffeine [2]. Caffeine has antioxidant, anti-inflammatory, liver protection, diabetes prevention, cardiovascular protection, weight loss, anticancer and neurodegenerative disease prevention and other health effects [3–9]. Nevertheless, the long-term or excessive intake of caffeine may cause insomnia, migraines, and changes in intraocular pressure [10]. Therefore, the creation of low and/or caffeine-free tea (caffeine <1%) has attracted extensive attention.

Similar to caffeine’s structure, multiple pharmacological studies have shown that theacrine has antidepressant, sedative-hypnotic, memory-improving, and nonalcoholic fatty liver-preventing effects [11–14]. However, tea plants containing theacrine are relatively scarce and the content is low. Yang *et al.* found that the content of theacrine (15.8 mg·g^−1^) was significantly higher than that of caffeine (9.4 mg·g^−1^) and theobromine (4.5 mg·g^−1^) in ‘Kucha’ (i.e. bitter tea), but it was not detected in *Camellia sinensis* and *C. ptilophylla* [15]. Previous studies demonstrated that the theacrine was synthesized from caffeine via being oxidized, isomerized, and methylated [16]. Recently, researchers identified three *N*-methyltransferases (NMTs) including a *N9*-methyltransferase (theacrine synthase, TcS) involved in purine alkaloids biosynthesis from Kucha leaves, which could catalyze the formation of theacrine from 1,3,7-trimethyluric acid [17]. However, the synthesis and regulatory mechanism of theacrine in tea germplasms rich in theacrine have not been clarified, and favorable alleles in specific resources have not been fully explored for genetic improvement.

In our previous study, we screened a tea germplasm, ‘Ruyuan Kucha’ (RY), from Ruyuan county, Guangdong province, which has high-level theacrine but low-level caffeine [2]. To understand the molecular mechanism for accumulating theacrine and innovating elite germplasm, we identified the key gene that results in the high-theacrine and low-caffeine trait of RY, clarified the molecular characteristics of allelic variation of *TcS*, and developed a functional marker to identify the favorable allele within RY and its offspring for greatly shortening the breeding period and improving the breeding efficiency. This study will facilitate for understanding the molecular genetic mechanism of the formation of high-theacrine and low-caffeine traits and provide the theoretical basis and technical support for the breeding of new cultivars with these elite traits.

## Results

### Variation of purine alkaloid content in F_1_ population

**Figure 1:**
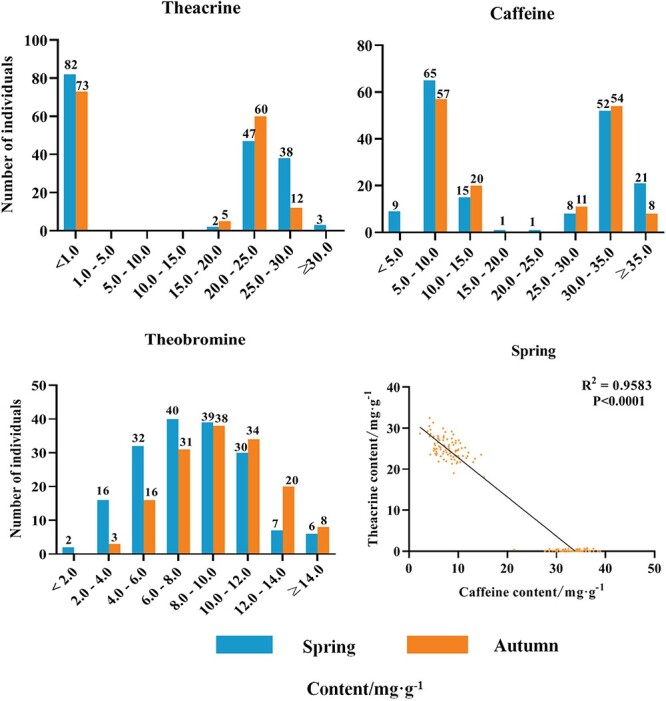
Distribution of purine alkaloids content in the ZC × RY F_1_ population. The vertical axis represents the number of individuals, and the abscissa represents the content of purine alkaloids. Correlation between theacrine and caffeine tested using Pearson’s correlation analysis.

The purine alkaloid in parents (‘Zhongcha 302’ (ZC) and RY) and their F_1_ individuals were determined by ultraperformance liquid chromatography (UPLC), and their variation and distribution are shown in Table 1 and Fig. 1. The theacrine content in RY was 21.3 mg·g^−1^ in spring and 23.7 mg·g^−1^ in autumn, but it was not detected in ZC. The theobromine content of F_1_ population was mainly distributed in the range of 5.0–12.5 mg·g^−1^. The theacrine content of 90 individuals in spring (*n* = 172) and 77 individuals in autumn (*n* = 150) was higher than 15.0 mg·g^−1^, and their caffeine content was lower than 20.0 mg·g^−1^. The theacrine content in remaining individuals was less than 1.0 mg·g^−1^, most of them were not detected, and their caffeine content was higher than 20.0 mg·g^−1^. The correlation analysis suggests a significant negative correlation between caffeine and theacrine (*P* < 0.0001). In spring, the offspring was expected to segregate as 1:1 for the 92 high theacrine individuals and 82 low theacrine individuals in the F_1_ population by chi-square test (χ^2^ = 0.37). These results indicate that the theacrine content was controlled by a single locus.

**Table 1:** The variation of purine alkaloid content of ZC × RY F_1_ population

Composition	Season	RY (mg·g^−1^)	ZC (mg·g^−1^)	F_1_ offspring				
				Range (mg·g^−1^)	Mean ± SD (mg·g^−1^)	CV (%)	Skewness	Kurtosis
Theacrine	Spring	21.32 ± 0.19	0.00 ± 0.00	0.00–32.49	13.22 ± 12.68	95.95	−1.93	−0.03
	Autumn	23.65 ± 0.06	0.00 ± 0.00	0.00–27.91	11.71 ± 11.49	98.14	−1.96	−0.01
Caffeine	Spring	12.41 ± 1.38	34.61 ± 0.34	2.24–38.89	20.03 ± 12.97	64.77	−1.86	0.10
	Autumn	8.81 ± 0.82	29.71 ± 0.36	5.71–37.92	20.07 ± 11.85	59.08	−1.90	0.08
Theobromine	Spring	11.80 ± 1.39	6.25 ± 0.06	0.87–16.18	7.82 ± 3.16	40.36	−0.29	0.16
	Autumn	16.30 ± 0.54	10.22 ± 0.11	2.25–16.65	9.36 ± 2.88	30.79	−0.33	0.06

### Identifying theacrine synthase gene (*TcS*) through BSR-seq

**Figure 2:**
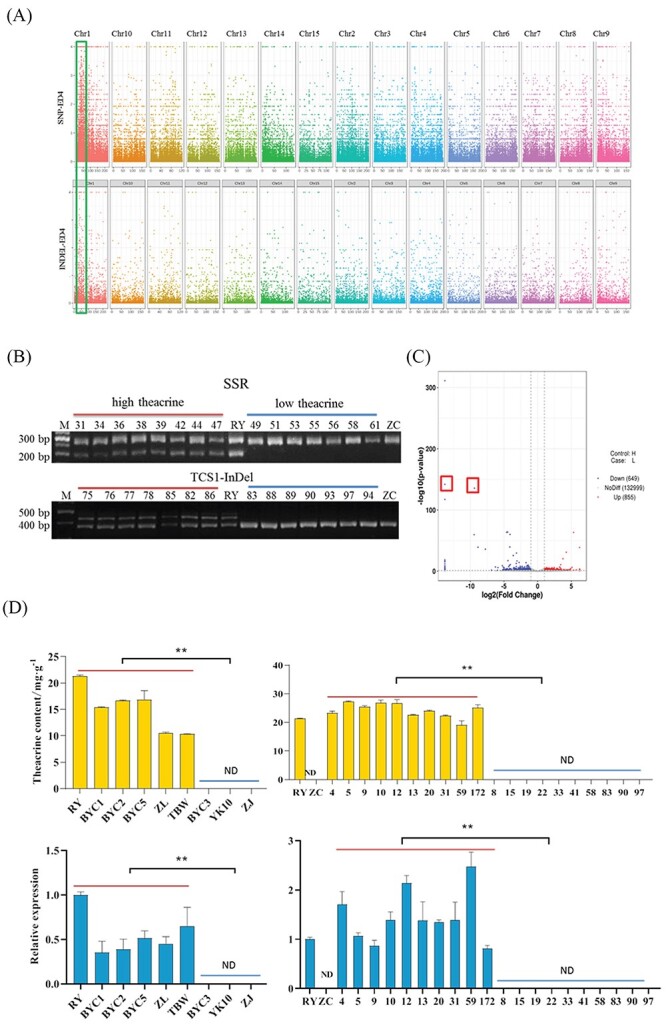
Identification of the key candidate genes that govern the high-theacrine and low-caffeine trait as determined by BSR-Seq and real-time PCR. (**A**) Chromosome distribution of ED4 values based on SNP and InDel. By using the chromosomal location of ‘Shuchazao’ genome as the abscissa and the ED value as the ordinate, the chromosomal scores of RNA-seq SNPs and InDel ED values were plotted. (**B**) SSR and InDel marker development validation. (**C**) Volcano plot of differentially expressed genes from no reference genome transcriptome sequencing; the conditions for screening differentially expressed genes were |log2FoldChange| > 1 and *P*-value <0.05. (**D**) Theacrine content and real-time PCR were used to analyse the *TcS* expression of different tea plants and individuals of F_1_ population. ^**^P<0.01.

To identify the candidate genes for controlling the theacrine content, we performed bulked segregant RNA sequencing (BSR-seq) by using two extreme pools (pool H/L). The theacrine content of individuals in each pool is shown in Table S2 (see online supplementary material). According to the distribution of ED values on the chromosome (Fig. 2A), we found an obvious peak surrounding 37 Mb on Chr1. Three of the 443 SNPs belonged to non-synonymous mutations in the transcript CSS0032602, which has high sequence identity (99.36%) with caffeine synthase gene (*TCS2*). Moreover, we developed molecular markers to confirm the linkage of high theacrine contents (Fig. 2B). The results show 250- and 200-bp DNA fragments in high-theacrine (>15.0 mg·g^−1^) individuals and only 250-bp DNA fragment in low-theacrine (<1.00 mg·g^−1^) individuals. The TCS1-InDel marker developed in our previous study [18] can also clearly distinguish the high-theacrine individuals.

Recently, Zhang *et al.* identified the key gene *CkTcS* for the theacrine synthesis from the transcriptome data of Kucha [17]. To further clarify the candidate genes for theacrine synthesis, the reads in the two pools were used for sequence splicing. The results show that two transcripts with significantly difference in mRNA-level (marked by red boxes) have high sequence identity (94.48% and 100%) with the reported *TcS* sequence (Fig. 2C and Table S3, see online supplementary material). Then, we amplified the cDNA sequence of *TcS* from RY and obtained 100% sequence similarity to the *CkTcS*.

Next, we investigated the transcript levels of *TcS* and theacrine contents in 30 accessions of tea germplasms with diverse genetic background. Intriguingly, we only amplified the cDNA sequence from eight tea accessions: ‘Zhongliu Kucha’ (ZL, from Jiangxi), ‘Tubawang’ (TBW, from Guangxi), ‘Baiyacha 1’ (BYC1), ‘Baiyacha 2’ (BYC2), ‘Baiyacha 3’ (BYC3), ‘Baiyacha 5’ (BYC5) (BYC1, BYC2, BYC3, and BYC5 from Fujian), ‘Yunkang 10’ (YK10) and ‘Zijuan’ (ZJ) (YK10 and ZJ from Yunnan). The theacrine contents in RY, BYC1, BYC2, BYC5, and ZL were higher than 10.0 mg·g^−1^, but were not detected in YK10, ZJ, and BYC3 (Fig. 2D). The quantitative real-time PCR (qRT-PCR) results show that the expression level of *TcS* in RY was higher than of that in other tea germplasms, and the expression level of BYC1, BYC2, and ZL were less than half of that in RY. Additionally, the expression levels of *TcS* in BYC3, YK10, and ZJ were not detected. Moreover, the qRT-PCR results show that the expression levels of *TcS* in high-theacrine F_1_ individuals were significantly higher than low-theacrine F_1_ individuals (Fig. 2D). These results suggest that *TcS* was the key candidate gene and the theacrine content was closely related to the expression level of *TcS*.

### Subcellular localization and *in vivo* functional identification of *TcS*

**Figure 3:**
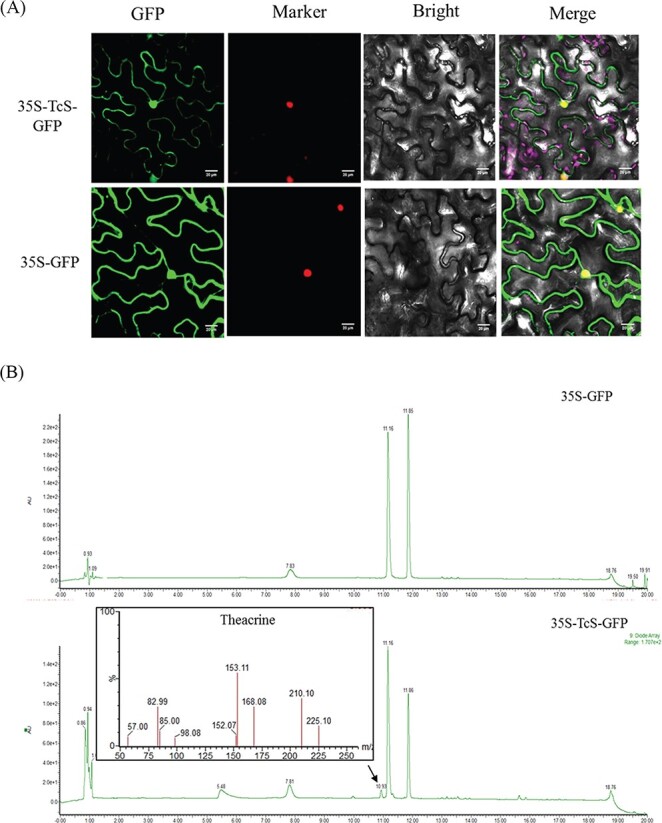
Subcellular localization and *in vivo* functional identification of *TcS*. (**A**) Subcellular localization of *TcS* in tobacco leaves. Bars = 20 μm. (**B**) LC–MS fragments of theacrine in the control and *TcS* transgenic tobacco treated leaves. Injection of 1,3,7-trimethyluric acid into T_0_ generation *TcS* transgenic tobacco leaves, transgenic tobacco with empty vector was used in the control group.

To validate the biological function of *TcS*, we firstly studied the subcellular localization of *TcS* using tobacco leaves, the results showing that the fluorescence signal of 35S-TcS-GFP was detected in the nucleus and cytoplasm (Fig. 3A). Zhang *et al.* found the *CkTcS* can catalyze the synthesis of theacrine from 1,3,7-trimethyluric acid *in vitro*, but the specific function of *TcS in vivo* remains to be studied [17]. Therefore, we injected 1,3,7-trimethyluric acid into *TcS*/EV transgenic tobacco leaves of T_0_ generation. The results show that the *TcS* transgenic plant can produce theacrine after absorbing 1,3,7-trimethyluric acid, but the control group cannot synthesize theacrine (Fig. 3B). Our results indicate that the *TcS* can catalyze theacrine synthesis *in vivo*.

### Analysis of *TcS* alleles and activity comparison of TcS recombinant enzyme

**Figure 4:**
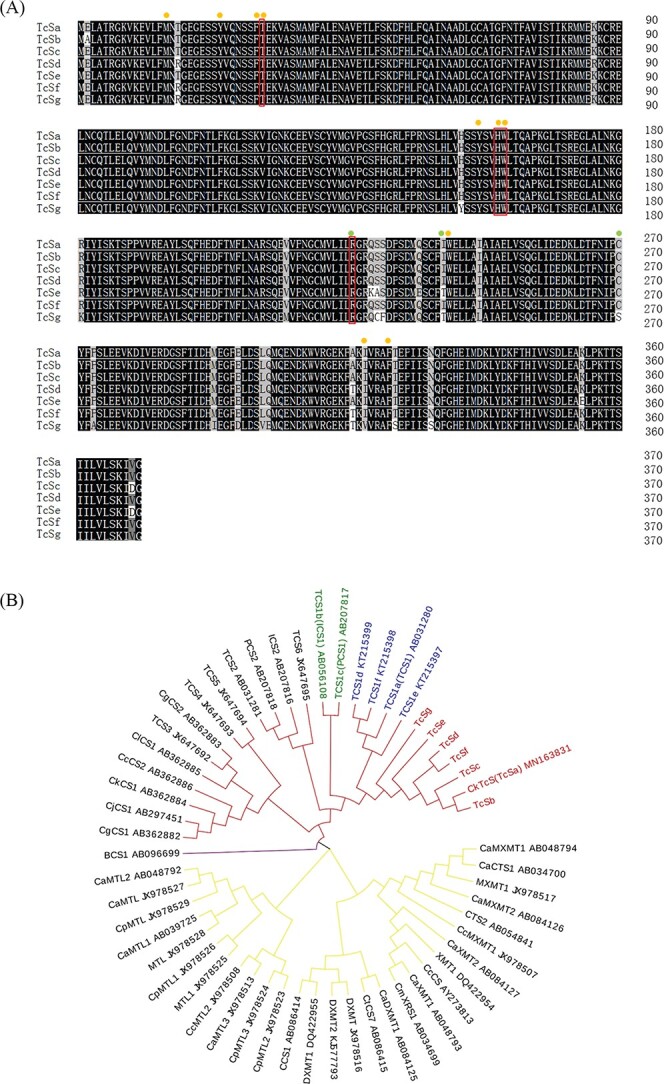
Comparison of amino acid sequences of TcSs and phylogenetic tree of *N*-methyltransferases. (**A**) Comparison of the amino acid sequences of TcS allelic variants. Residues liming the 1,3,7-trimethyluric acid binding pocket are highlighted by the yellow (conserved residues) and green dots (variable residues); residues that form direct hydrogen bond with 1,3,7-trimethyluric acid are labeled with a red box. (**B**) Phylogenetic tree of *N*-methyltransferases coding region sequences related to purine alkaloids in *Camellia*, *Theobroma*, and *Coffea* generated using the MEGA Program. Red, yellow and purple branches mark *NMTs* of *Camellia*, *Theobroma*, and *Coffea*. The green, blue, and red branches indicate TS*,* CS, and TcS.

To clarify the allelic variation of *TcS* among different tea germplasms, we amplified cDNA sequence from five tea accessions. A total of seven *TcS* alleles were obtained and were named as *TcSa* (*CkTcS*), *TcSb*, *TcSc*, *TcSd*, *TcSe*, *TcSf*, and *TcSg* (Table S4, see online supplementary material). The coding regions of seven *TcS* alleles were all 1113 bp and encode 370 amino acids (Table S5, see online supplementary material). The similarities of nucleotide and amino acid sequence were more than 94% (Table S6, see online supplementary material). The comparison of amino acid sequences of TcSs revealed that key amino acid residues (Ile-241, Cys-270, Ile-318) in TcSe and TcSg were different with TcSa (Fig. 4A). Phylogenetic tree analysis was constructed to investigate the homology of NMTs from *Camellia*, *Coffea*, and *Theobroma* (Fig. 4B). The results show that NMTs in *Camellia*, *Coffea*, and *Theobroma* clustered into three independent groups, and the TCS1s and TcSs in tea plants were clustered into three discriminative branches, namely, theobromine synthase (TS), caffeine synthase (CS), and TcS. The TcS alleles cloned were clustered in the TcS class, which was clearly distinguished from CS and TS.

**Figure 5:**
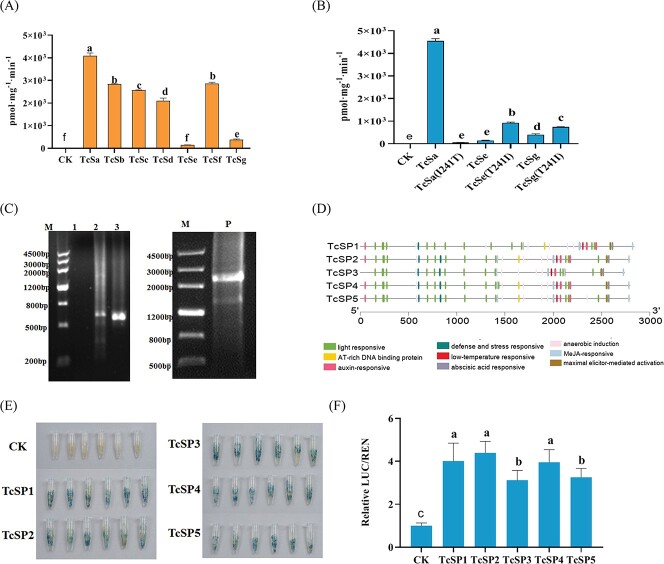
Comparison of the enzymatic and promoter activities of the TcS allelic variation. (**A**) Comparison of the enzymatic activity of TcSs (*n* = 3). CK: pMAL-c5x, *P* < 0.05; (**B**) Enzymatic activity of mutation protein (*n* = 3); (**C**) 536-bp sequence fragment obtained by genome walking and full-length fragment of *TcS* promoter with a length of 2686 bp on the right. 1–3: first-, second-, and third-round PCR product; P: *TcS* promoter. (**D**) Distribution of *cis*-acting element on the *TcS* promoter sequence of different variants. (**E**) GUS histochemical assay of different *TcS* promoters. (**F**) Dual-luciferase of different *TcS* promoter. Determination of the differences in promoter activity by detecting the ratio of firefly luciferase and renilla luciferase activities (*n* = 10). Different lowercase letters on the figure indicate significant difference at *P* < 0.05.

To elucidate the protein activity of TcSs, we analysed the enzymatic activity of recombinant proteins *in vitro* (Fig. 5A). The results showed that all TcSs had *N*9-methylation activities, and their activity varied greatly. The TcSa had the highest enzymatic activity among seven alleles, and no significant difference was observed between TcSb and TcSf. The enzymatic activities of both TcSe and TcSg were the lowest due the T241I mutation, and the activities were only 3.57% and 9.52% of TcSa, respectively. The effect of 241st amino acid residue was verified by mutating the Thr-241 of TcSa to Ile-241 and the Ile-241 of TcSe and TcSg to Thr-241. The results show that enzyme activity of TcSe(T241I) was 6.92 times of TcSe, and the enzyme activity of TcSg(T241I) was 1.89 times of TcSg, while the enzyme activity of TcSa(I241T) was extremely low (50.68 pmol·mg^−1^·min^−1^) and significantly lower than TcSa (Fig. 5B). These results demonstrate that 241st amino acid residue could significantly affect the TcS activity.

### Activity analysis of the different *TcS* promoters and development of functional marker

The foregoing results found that both RY and BYC3 contained *TcSa* allele, but we cannot detect the theacrine in BYC3. Therefore, we speculate that this is probably due to the transcription of *TcSa* being inhibited in BYC3. To verify this assumption, using the genomic DNA of RY as a template, a 536 bp *TcS* promoter fragment was obtained by genome walking. Then, PCR amplification was used to obtain a sequence with a length of 2686 bp (Fig. 5C). The TcS promoters of RY, BYC1, BYC3, ZL, and TBW were further amplified and named as TcSP1, TcSP2, TcSP3, TcSP4, and TcSP5, respectively. Their sequence lengths were 2831, 2794, 2737, 2793, and 2794 bp (Fig. 5D**,**Table S7, see online supplementary material). The sequence alignment shows that TcSP1, TcSP2, TcSP3, TcSP4, and TcSP5 were highly similar (80.70%–99.79%, Table S8, see online supplementary material). In comparison with TcSP2, TcSP3, TcSP4, and TcSP5, TcSP1 has a 234-bp deletion between −503 and −270 (with the start codon ATG as the starting site) and a 271-bp insertion between −1654 and −1384.

The *cis*-acting element prediction indicates that *TcS* promoters had a lot of light-responsive, MeJA-responsive, abscisic acid responsive, auxin responsive, and anaerobic induction elements (Fig. 5D). Except that BYC3 lacks the AT-rich DNA-binding protein element, all the *TcS* promoters have an AT-rich DNA-binding protein and a maximal elicitor-mediated activation element distributed at the 3′ flanking of the promoter. The 271-bp insertion in RY have several CAAT-box, TATA-box, CARE, and Unnamed *cis*-acting elements. The result of GUS histochemical analyses indicated that TcSP1-TcSP5 could initiate the GUS expression of downstream (Fig. 5E). Dual luciferase assay shows that the activities of TcSP1, TcSP2, and TcSP4 had no significant differences, but were significantly higher than those of TcSP3 and TcSP5 (Fig. 5F). Although certain differences were observed between different promoters, the mechanism of change in the content of theacrine still needs to be further studied.

**Figure 6:**
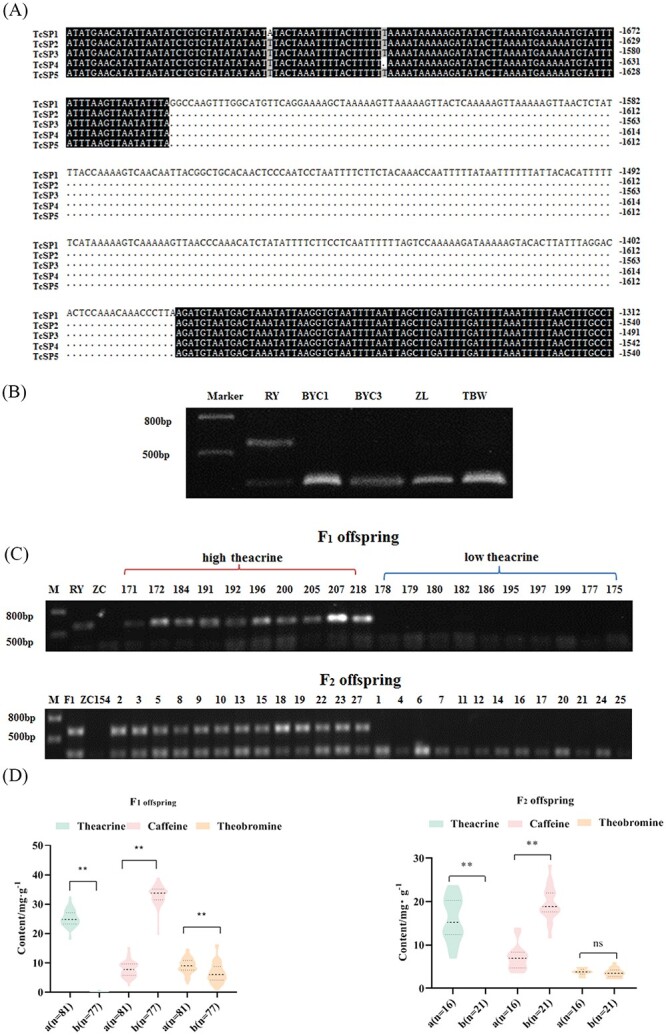
Development and verification of a functional marker based on the 271-bp insertion of RY’s promoter. (**A**) *TcS* promoter sequence alignment. The 271-bp insertion sequence of TcSP1 in RY. (**B**) TcS-InDel amplification results in different tea germplasms. (**C**) TcS-InDel amplification results of F_1_ offspring and F_2_ offspring. F_1_ offspring parents: RY and ZC; F_2_ offspring parents: a F_1_ offspring and ZC154. (**D**) The contents of purine alkaloids with target band were amplified by TcS-InDel marker in F_1_ offspring of F_2_ offspring. ^**^P<0.01.

To accelerate the breeding cycle for high-theacrine tea plants, we developed an InDel marker based on the promoter region of RY. The results show that two DNA fragments were amplified in RY, while only one DNA fragment was amplified in other germplasms (Fig. 6B). Consistant with their parents, the 604-bp fragment could be amplified in RY and high-theacrine individuals, but it did not exist in ZC and low-theacrine individuals (Fig. 6C and D). Moreover, we tested this marker in a F_2_ population by crossing ‘Zhongcha 154’ (ZC154) with a high-theacrine individual of F_1_ population. Among them, 16 individuals containing theacrine had a 604-bp band identified by TcS-InDel marker. These results suggest that our molecular marker could be used for selecting high/low-theacrine individuals of RY’s offspring.

## Discussion

### Identification of the key gene governing the high-theacrine and low-caffeine traits

Although important progress has been made in the study of the key gene (*TcS*) related to theacrine metabolism, the function and molecular regulatory mechanism of *TcS* has not been fully verified, and genetic evidence remains lacking to support their role in regulating the formation of the high-theacrine trait, which cannot be directly applied to the genetic improvement. The progress of high-throughput sequencing technology and the publication of a chromosome level reference map of the tea plant genome [19–21] provide an important technical basis for further exploring the favorable genes of theacrine. In the present study, we constructed a F_1_ population crossing by ZC and RY, and the F_1_ offspring was expected to segregate for the high theacrine individuals and low theacrine individuals as 1:1. The BSR-Seq analysis of the individuals with obvious differences in theacrine content of F_1_ population revealed surrounding the 37 Mb region of Chr1 was most highly correlated to the theacrine trait compared with the reference genome of ‘Shuchazao’. The localization results were confirmed by a SSR marker surrounding 37 Mb and an InDel marker based on *TCS1* sequence. The TcS-InDel marker was co-segregated with high-theacrine and low-caffeine traits, and *TcS* genotype can explain more than 95% and 97% of the phenotypic variation of caffeine and theacrine in two seasons, respectively (Table S9, see online supplementary material). Real-time PCR indicated that theacrine content was determined by the expression level of *TcS*. Moreover, the function of *TcS* in plants was identified by stably genetic transformed tobacco and verified *in vivo*. In conclusion, our study identifies *TcS* is the key gene that governs the high-theacrine and low-caffeine traits.

Most of the amplification of *NMTs* related to purine alkaloid biosynthesis was expanded through the most recent gene duplication events [22, 23]. In the present study, *TcS* was found only in a few tea germplasms and not amplified in *Camellia sinensis var. sinensis*, which further supports the independent, recent, and rapid evolution of the tea plant.

### Identification a favorable *TcS* allele from RY with potential use in breeding

Plants have a large number of allelic variations within the population, causing phenotypic changes. In the present study, *TcS* alleles (*TcSa*-*TcSg)* were cloned from different tea germplasms from Jiangxi (ZL), Guangdong (RY), Fujian (BYC1 and BYC3), and Guangxi (TBW). All TcS alleles could catalyze the theacrine synthesis from 1,3,7-trimethyluric acid *in vitro*. Among these alleles, TcSa had the highest enzyme activity, but enzyme activities of TcSe and TcSg were only 3.57% and 9.52% of TcSa. The enzyme activity of single mutation T241I on TcSe and TcSg were remarkably enhanced, while the enzyme activity of TcSa(I241T) remarkably decreased compared with TcSa. By using site-directed mutagenesis experiments, the 241st amino acid residue was verified as the key residue, and it significantly affected TcS activity. Promoters can regulate gene transcription and significantly affect gene expression. Both GUS histochemical analyses and dual-luciferase assay method all confirmed that different *TcS* promoters could effectively drive foreign gene expression and the promoter activity of TCSP1 was relatively high in RY. Moreover, RY’s offspring containing *TcS* in F_1_ and F_2_ population all accumulated high content of theacrine. Thus, the novel *TcS* allele from RY is a favorable allele and provides a valuable genetic resource for high-theacrine breeding.

Side effects, especially in children, adolescents, pregnant women, and people who are sensitive to caffeine, should reduce or limit caffeine intake to avoid potential adverse effects [24]. Many researchers have cultivated low-caffeine tea plant through cross-breeding, grafting, and biotechnology, but the effectivity was low and unstable [25--27]. Interestingly, the caffeine content decreased with increasing theacrine content. Thus, wild tea plants with high theacrine trait may become a valuable and potential resource for decreasing caffeine content in cultivated tea plants. Simultaneously, this study identified nine individuals in spring with caffeine content lower than 5.0 mg·g^−1^ in ZC × RY F_1_ population. A lower content of caffeine can be obtained by aggregating two alleles by crossing with two individuals of RY’s offspring in future. Our research provides a new technology path, elite gene resource, and the possibility for stable cultivation of low-caffeine tea plants.

### A functional marker with powerful use in the genetic improvement of purine alkaloid

Tea plant is a woody perennial crop with long generation intervals. At present, a stable genetic transformation system and gene editing technology have not been established for directional genetic improvement. Some problems are encountered in the genetic improvement of theacrine and caffeine traits by conventional breeding, such as blind parental selection, long identification cycle, and high cost. To address these bottlenecks, we developed molecular markers based on a 271-bp insert between −1654 and −1384 of TcSP1 in RY. TcS-InDel marker was developed according to the difference of *TcS* promoter sequences, which can clearly identify all high-theacrine and low-caffeine individuals in the RY’s offspring and verify the individuals containing theacrine at the seedling stage. Therefore, the TcS-InDel, which was developed based on the gene sequence, can be used as a functional marker for future high-theacrine and low-caffeine tea plant cultivation to improve the efficiency and accuracy of breeding and facilitates the rapid identification and screening of genetic materials.

## Materials and methods

### Plant materials

In this study, we used a F_1_ population constructed by the ZC crossed with RY. The F_2_ offspring was innovated by the hybridization of ZC154 and a high-theacrine individual of ZC × RY F_1_ population. Eight accessions of tea germplasms (ZL, TBW, BYC1, BYC2, BYC3, BYC5, YK10, and ZJ) containing *TcS* were used as plant materials to isolate and identify alleles. These tea resources were collected from their original regions, and then grown at the Tea Research Institute of the Chinese Academy of Agricultural Sciences located at Hangzhou, Zhejiang, China. The tea samples were vacuum-freeze-dried for 4 days. The fresh tea samples were transported in liquid nitrogen and stored at −80°C for DNA and RNA extraction.

### Sample preparation and UPLC conditions

Young shoots were obtained from parents and individuals in F_1_ and F_2_ populations. The powdered freeze-dried tea samples (0.1000 ± 0.0003 g) were extracted with 10 mL of 70% (v/v) methanol aqueous solution, sonicated for 30 min, and then stored at 4°C for 2 h. The supernatant was filtered through a 0.22-μm nylon filter into a brown injection bottle and stored at −80°C for the following experiments. The UPLC conditions employed by Liu *et al.* were used [28].

### Identification of candidate gene by BSR-Seq

The bulked groups consisting of 18 high- (≥15 mg·g^−1^) and 18 low- (<1 mg·g^−1^) theacrine individuals of ‘one and a bud’ were selected from the ZC × RY F_1_ population for BSR-seq. The theacrine content is shown in Table S2 (see online supplementary material). Total RNA was extracted following the manufacture’s protocol of the EASY-spin Plus Complex Plant RNA kit (Aidlab Biotechnologies Company, Beijing, China). The RNA library was sequenced using second-generation sequencing technology and Illumina’s sequencing platform, and the statistical data were collected. The base quality, base content (AT, GC separation phenomenon), GC distribution, and average quality of the data were tested to further obtain high-quality data. SNP and InDel were detected and annotated by using the latest ‘Shuchazao’ genome [19] as reference to locate the candidate gene. In the absence of a reference genome, the high-quality read was spliced to obtain unigene for further analysis of the gene function and expression.

### Gene expression analysis by real-time PCR

Total RNAs were extracted following the manufacture’s protocol of the EASY-spin Plus Complex Plant RNA kit (Aidlab Biotechnologies Company, Beijing, China), estimated using a NanoDrop ultraviolet spectrophotometer (Thermo, Waltham, MA, USA), and diluted to 100 ng·μL^−1^.Based on the instructions of the FastKing cDNA First-Strand synthesis kit (TIANGEN, Biotechnologies Company, Beijing, China), 2 μL of RNA was used to synthesize first-strand cDNA, and then dilute it to a total volume of 200 μL.

qRT-PCR reaction was caried out using the LightCycler® 480 System with the LightCycler 480 SYBR Green I Master and in the reaction system (10 μL) consisting of 5 μL of SYBR Green Master Mix, 0.4 μL of forward/reverse primers, 2 μL of cDNA, and 2.2 μL of sterile water. The procedure involves the following steps: 45 cycles of pre-denaturation at 94°C for 10 s, denaturation at 94°C for 10 s, annealing at 58°C for 15 s, and extension at 72°C for 12 s. The relative gene expression was calculated using the 2^−ΔΔCT^ method.

### Subcellular localization of *TcS*

*TcS* without stop codon ‘TAG’ was cloned into 35 s-GFP vector. The constructed vector plasmid was transferred into Agrobacterium GV3101 by using the heat shock method and cultured at 28°C for 2 days. Five clones were selected from the overnight culture plate into 1 mL of LB liquid medium (50 ng·mL^−1^ kanamycin, 25 ng·mL^−1^ rifampicin) and cultured in a shaker at 28°C and 200 rpm for 2–3 h. Positive clones were screened via bacterial liquid PCR and sent to Shanghai Huajin Biotechnology Co., Ltd for sequencing verification. Approximately 100 μL of *Agrobacterium tumefaciens* were transferred into 10 mL of liquid LB medium (containing 25 ng·mL^−1^ of rifampicin and 50 ng·mL^−1^ of kanamycin) to expand to an OD_600_ value of 1.0–1.2. The pellets were collected by centrifugation at 4000 rpm and 4°C for 10 min. After culturing in the dark for 2–3 h at room temperature, it was then injected into tobacco leaves.

After the normal cultivation of transgenic tobacco for 2–3 days, the subcellular location was observed using a confocal laser scanning microscope. The experimental control group was transgenic tobacco with empty vector, and DAPI marker was used as the co-localized marker.

### Validation function by transgenic tobacco of *TcS*

*TcS* was cloned into the 35 s-GFP vector, and then transformed into GV3101. When wild-type tobacco seeds were grown to 3–4 leaves, sterile tobacco leaves were cut into 0.5 cm × 0.5 cm sections with a scalpel, and the explants were infected. The *Agrobacterium* suspension containing the stop codon-containing TcS-GFP fusion expression vector was immersed in the explants for 10 min, followed by co-cultivation, and cultured in the dark at 25°C for 2 days to differentiate and screen the seedlings. Differentiation was carried out for 30–40 days, while the rooting time was 10–15 days. The DNA of tobacco leaves was extracted for verification, the correct transgenic tobacco was planted in nutrient soil, 0.4 mM of 1,3,7-trimethyluric acid was injected into the back of the transgenic tobacco leaves, and samples were obtained after 24 h. Finally, liquid chromatography-mass spectrometry (LC–MS) [29] was used to detect its components, and the experimental control group used transgenic tobacco with empty vector.

### Amplification of the full-length cDNA and promoter of *TcS* allelic variants

By using RY cDNA as a template, *TcS* was amplified using primer TcSF/R, and PCR was performed in a final reaction volume of 50 μL by using KOD-Plus-Neo (Toyobo, Japan). The PCR conditions were as follows: 94°C for 2 min, 35 cycles of 94°C denaturation for 10 s, annealing at 58°C for 25 s, extension at 68°C for 30 s, and extension at 68°C for 5 min. Then, DNA sequencing was performed by Shanghai Huajin Biological Company. By using RY genomic DNA as a template, based on the 5′ UTR of *TcS* by transcriptome sequencing, three specific primers SP1, SP2, and SP3 were used to amplify the *TcS* promote sequence. The three-round PCR conditions were based on the genome walking kit (ZOMAN Biotechnologies Company, Beijing, China), and the PCR products were identified by 1% agarose gel electrophoresis and sequenced Shanghai Huajin Biological Co., Ltd.

### Protein purification and activity assay of recombinant TcS

The cDNA encoding *TcS* allelic variants were cloned into a pMAL-c5x vector (NEB, Beijing, China) containing the recombinant maltose binding protein by using the primer TcSpMALF/R. The plasmids were verified by sequencing, and then transformed into *BL21*(DE3) *pLys* cells (TransGen Biotechnologies Co., Beijing, China). The fusion expression of the target gene in *Escherichia coli*, the detection of the target protein, and the quantification of the purified protein were carried out as described by Jin *et al.* [18].

The purified protein induced by empty vector was used as the control group. The enzyme reaction mixture consisted of 100 mM MES (pH = 6.5), 0.2 mM MgCl_2_, 0.5 mM SAM, 0.2 mM 1,3,7-trimethyluric acid, and 20 μg of purified protein, and ddH_2_O was added to a volume of 200 μL. The mixture was incubated at 30°C for 1 h, and 200 μL of 0.1 M HCl solution was added to stop the reaction. The mixture was centrifuged at 12000 rpm for 2 min, and then filtered with a 0.22-μm nylon filter into a brown liquid phase bottle. Each protein was determined thrice in duplicate. The TcS allelic variants activity was determined by HPLC as described by Jin *et al.* [18].

### Promoter activity assay of *TcS*

The promoter activity assay consists of the tobacco expression GUS staining assay and tobacco dual-luciferase reporter assay. *TcS* promoter sequence was recombined into PBI1101.3-GUS plus vector. Then, the vector containing the target sequence was infected with tobacco leaves, and after 3 days of cultivation, tobacco leaves were obtained for GUS staining experiment. This GUS staining experiments were conducted based on the instructions of GUS staining kit (Gcloning Co., Ltd, Beijing, China).

*TcS* promoter sequence was recombined into pGreenII0800-Luc vector. Then, the plasmids verified by sequencing were transformed into GV3101 (pSoup-19). When the GV3101 cell was transformed to tobacco leaves by agroinfiltration, the experiment and control group were injected into 10 pots of tobacco leaves and incubated after 3 days. The dual-luciferase activity assay was carried out based on the Dual-Glo® Luciferase assay system kit (Promega Biotechnologies Co., Beijing, China).

### Marker development

Gene markers were developed based on the specificity of the *TcS* promoter sequence and full-length gene sequence in RY. Based on the upstream and downstream sequences of this sequence (between −1804 and −1201), a pair of primers TcS-InDelF and TcS-InDelR was designed for PCR. PCR products were identified by 1% agarose gel electrophoresis.

## Statistical analyses

Data are presented as mean ± standard error (SD). Statistical significance of differences between two groups was determined by Student’s *t*-test and statistical significance of differences among groups was determined with Tukey’s test using SPSS software (SPSS, Chicago, IL, USA). Pearson correlation analysis was used to test the correlation of theacrine and caffeine content in the F_1_ offspring, and graphing was performed using GraphPad Prism 8.

## Supplementary Material

supp_data_uhac191

## Data Availability

All data supporting the findings of this study are available within the paper and within its supplementary materials published online. Raw sequences and FASTA files of BSR-Seq can be found at NCBI BioProject: PRJNA853132.
