# Supplementary material for: A novel TcS allele conferring the high-theacrine and low-caffeine traits and having potential use in tea plant breeding
Source: Hortic Res. 2022 Aug 25;9:uhac191. doi: 10.1093/hr/uhac191 (PMC9630966; doi:10.1093/hr/uhac191)
Supplement: supp_data_uhac191 [file supp_data_uhac191.zip › 6.29 Supplementary Table S1-9.docx]

**Table S1.** Primer sequences used in this study

| Primer name | Primer sequence（5ʹ-3ʹ） | |  |
| --- | --- | --- | --- |
| 18S-F | TCTCAACCATAAACGATGCCGACCAG | |  |
| 18S-R | TTTCAGCCTTGCGACCATACTCCC | |  |
| TcSF | TAAAAATTACTTTTCTGACGAGGCA | |  |
| TcSR | CYGCTGCGTTAATAGCTTGAAAAAG | |  |
| SSR-F | ACAGCGACTCAATATGATGGT | |  |
| SSR-R | TGCACCCCTATACATATCATACGC | |  |
| TCS1-InDelF | TATGTCATGTTTCTATTATTT | |  |
| TCS1-InDelF | TACTTTCTCCTTCTCCTCTGT | |  |
| TcSFcDNA | TCGCGAATGCATCTAGATGCTGGCCTCTTTGATAAA | |  |
| TcSRcDNA | ACGGGCCCGGGATCCGATCTATCCAACAATCTTGGAAA | |  |
| TcSpMALF | GCGATATCGTCGACGGATCCATGGAG/CGCTAGCTACTAGGGG | |  |
| TcSpMALR | TAATTACCTGCAGGGAATTCCTATCCAAC/TCAATCTTGGAAA | |  |
| SP1 | CAACACTTCTTTCACCTTCCCCCT | |  |
| SP2 | TTCTTTCACCTTCCCCCTAGTAGC | |  |
| SP3 | CTGCCACAGCGGTGATATGAATAT | |  |
| TcSPF | TCGCGAATGCATCTAGATGTAG/AGGTCAAGAATGTGGGTT | |  |
| TcSPR | ACGGGCCCGGGATCCGATTTCACCTTCCCCCTAGTAGC | |  |
| TcSP (LUC)F | CTATAGGGCGAATTGGGTACCGAGTA/GTAGGTCAAGAATGTGGGTTTAT | |  |
| TcSP (LUC)R | GTTTTTGGCGTCTTCCATGGGCCTCGTCAGAAAAGTAATTTT | |  |
| TcSP (GUS)F | ATGCCTGCAGGTCGACGTAG/AGGTCAAGAATGTGGGTTTAT | |  |
| TcSP (GUS)R | CTCAGATCTACCATGGTACCGCCTCGTCAGAAAAGTAATTTTTAT | |  |
| TcS-InDelF | AGGTACATACGATACATACC | |  |
| TcS-InDelR | | TTGAGATTCACTTACTGAGT | |

**Table S2.** High-theacrine individuals (pool H) and low-theacrine individuals (pool L) for BSR-seq

| Pool H | individual | theacrine content  （mg·g^-1^） | Pool L | individual | thecrine content  （mg·g^-1^） |
| --- | --- | --- | --- | --- | --- |
| H1 | 3 | 28.09 | L1 | 8 | 0.00 |
|  | 4 | 23.30 |  | 15 | 0.74 |
|  | 5 | 27.27 |  | 19 | 0.00 |
|  | 7 | 24.83 |  | 23 | 0.00 |
|  | 12 | 27.04 |  | 33 | 0.00 |
|  | 18 | 29.62 |  | 35 | 0.00 |
| H2 | 20 | 24.05 | L2 | 41 | 0.00 |
|  | 26 | 27.04 |  | 46 | 0.00 |
|  | 39 | 22.41 |  | 48 | 0.00 |
|  | 44 | 24.82 |  | 49 | 0.00 |
|  | 59 | 19.04 |  | 55 | 0.00 |
|  | 70 | 28.09 |  | 58 | 0.00 |
| H3 | 91 | 24.59 | L3 | 66 | 0.00 |
|  | 96 | 24.27 |  | 72 | 0.00 |
|  | 98 | 24.50 |  | 80 | 0.00 |
|  | 102 | 28.45 |  | 81 | 0.00 |
|  | 117 | 24.83 |  | 90 | 0.00 |
|  | 119 | 25.85 |  | 94 | 0.00 |

**Table S3.** Differential expression results of transcripts

| ID | H | L | foldchange（L/H）） | log2FoldChange | P value |
| --- | --- | --- | --- | --- | --- |
| TRINITY_DN75399_c0_g1 | 5461.46 | 8.17 | 0.0014 | -9.36 | 2.33E-136 |
| TRINITY_DN3025_c1_g1 | 1649.76 | 0 | 0 | -Inf | 1.12E-142 |

| >TRINITY_DN75399_c0_g1  AAAGAAATGCAGGGAATTGAATTGCCAAACACTGGAACTTCAGGTTTACATGAATGATCTTTTTGGAAACGATTTCAATACCCTCTTCAAAGGCCTGTCGTCTAAGGTTATTGGTAACAAATGCGAGGAAGTTTCTTGTTATGTGATGGGAGTACCAGGGTCTTTCCATGGCCGGCTTTTTCCTCGTAACAGCTTGCATTTAGTTCATTCGTCTTACAGTGTTCATTGGCTTACTCAGGCACCAAAAGGACTCACAAGTAGAGAAGGCTTGGCATTAAACAAGGGGAGGATTTACATATCAAAGACAAG |
| --- |
| >TRINITY_DN3025_c1_g1  TCTCCAAAGATTTCCACCTTCTTCCAGCTCTTAATGCAGCGGACTTGGGTTGTGCAGCGTGTCCAAACACATTCGCAGTGATTTATACGATCAAGAGAATGATGGAAAATAAATGCAGGGAATTGAATTGCCAATCACTGGAACTTCAGGTTTACTTGATTGATCTTTTTGGAAATGATTTCAAGACCCTCTTCAAAGGCCTGTCGTCTAAGGTTATTGGTAACAAATGCGAGGAAGTTTCTTGTTATGTGATGGGAGTACCAGGGTCTTTCCATGGCCGGCTTTTTACTCGTAACAGCTTGCATTTAGTTCTTTCCTCTTACAGT |

**Table S4.** TcS allelic variation in different tea resources

| Tea germplasms | theacrine content（mg·g^-1^） | TcS alleles |
| --- | --- | --- |
| RY | 21.32±0.03 | TcSa（TcS） |
| ZL | 10.70±0.04 | TcSb, TcSc |
| BYC1 | 15.40±0.03 | TcSd |
| BYC3 | ND | TcSa, TcSe |
| TBW | 10.40±0.02 | TcSf, TcSg |

**Table S5.** Coding region nucleotide sequence of *TcS* allelic variation in different tea resources

| >CkTcS(TcSa)  ATGGAGCTAGCTACTAGGGGGAAGGTGAAAGAAGTGTTGTTCATGAACACAGGAGAAGGAGAAAGTAGTTATGTACAAAACTCTTCTTTCACAGAAAAAGTGGCCTCAATGGCAATGCCAGCGCTAGAAAATGCAGTTGAAACTCTCTTCTCCAAAGATTTCCACCTTTTTCAAGCTATTAACGCAGCAGACTTGGGTTGTGCAACGGGTCCAAACACGTTCGCAGTGATTTCTACGATCAAGAGAATGATGGAAAAGAAATGCAGGGAATTGAATTGCCAAACACTGGAACTTCAGGTTTACATGAATGATCTTTTTGGAAACGATTTCAATACCCTCTTCAAAGGCCTGTCGTCTAAGGTTATTGGTAACAAATGCGAGGAAGTTTCTTGTTATGTGATGGGAGTACCAGGGTCTTTCCATGGCCGGCTTTTTCCTCGTAACAGCTTGCATTTAGTTCATTCGTCTTACAGTGTTCATTGGCTTACTCAGGCACCAAAAGGACTCACAAGTAGAGAAGGCTTGGCATTAAACAAGGGGAGGATTTACATATCAAAGACAAGCCCTCCTGTTGTAAGAGAAGCCTACTTATCTCAATTTCATGAAGATTTCACAATGTTTCTTAATGCTAGATCCCAAGAGGTGGTTCCAAATGGTTGTATGGTGTTGATACTTCGTGGTAGGCAATCTTCTGATCCTTCAGACATGCAGAGCTGCTTTATTTGGGAACTATTAGCTATAGCCATTGCTGAATTGGTTTCGCAGGGATTGATAGATGAAGATAAATTAGACACCTTCAATATACCTTGCTATTTTCCATCACTTGAGGAAGTGAAAGATATAGTGGAGAGGGACGGATCATTCACAATTGATCATATGGAGGGGTTTGAACTTGATAGCCTACAGATGCAAGAGAATGATAAATGGGTTAGAGGGGAAAAGTTTGCCAAGATTGTCAGGGCCTTCACAGAGCCTATAATTTCAAACCAGTTTGGACATGAAATCATGGACAAACTATATGACAAATTCACTCACATTGTAGTTTCAGATTTGGAAGCAAAGCTGCCGAAGACCACAAGTATCATCCTAGTGCTTTCCAAGATTGTTGGATAG |
| --- |
| >TcSb  ATGGCGCTAGCTACTAGGGGGAAGGTGAAAGAAGTGTTGTTCATGAACACAGGAGAAGGAGAAAGTAGTTATGTACAAAACTCTTCTTTCACAGAAAAAGTGGCCTCAATGGCAATGCCAGCGCTAGAAAATGCAGTTGAAACTCTCTTCTCCAAAGATTTCCACCTTTTTCAAGCTATTAACGCAGCAGACTTGGGTTGTGCAACGGGTCCAAACACGTTCGCAGTGATTTCTACGATCAAGAGAATGATGGAAAAGAAATGCAGGGAATTGAATTGCCAAACACTGGAACTTCAGGTTTACATGAATGATCTTTTTGGAAACGATTTCAATACCCTCTTCAAAGGCCTGTCGTCTAAGGTTATTGGTAACAAATGCGAGGAAGTTTCTTGTTATGTGATGGGAGTACCAGGGTCTTTCCATGGCCGGCTTTTTCCTCGTAACAGCTTGCATTTAGTTCATTCGTCTTACAGTGTTCATTGGCTTACTCAGGCACCAAAAGGACTCACAAGTAGAGAAGGCTTGGCATTAAACAAGGGGAGGATTTACATATCAAAGACAAGCCCTCCTGTTGTAAGAGAAGCCTACTTATCTCAATTTCATGAAGATTTCACAATGTTTCTTAATGCTAGATCCCAAGAGGTGGTTCCAAATGGTTGTATGGTGTTGATACTTCGTGGTAGGCAATCTTCTGATCCTTCAGACATGCAGAGCTGCTTTATTTGGGAACTATTAGCCATAGCCATTGCTGAATTGGTTTCGCAGGGATTGATAGATGAAGATAAATTAGACACCTTCAATATACCTTGCTATTTTCCATCACTTGAGGAAGTGAAAGATATAGTGGAGAGGGACGGATCATTCACAATTGATCATATGGAGGGGTTTGAACTTGATAGCCTACAGATGCAAGAGAATGATAAATGGGTTAGAGGGGAAAAGTTTGCCAAGATTGTCAGGGCCTTCACAGAGCCTATAATTTCAAACCAGTTTGGACATGAAATCATGGACAAACTATATGACAAATTCACTCACATTGTAGTTTCAGATTTGGAAGCAAAGCTGCCGAAGACCACAAGTATCATCCTAGTGCTTTCCAAGATTGTTGGATAG |
| >TcSc  ATGGAGCTAGCTACTAGGGGGAAGGTGAAAGAAGTGTTGTTCATGAACACAGGAGAAGGAGAAAGTAGTTATGTACAAAACTCTTCTTTCACAGAAAAAGTGGCCTCAATGGCAATGCCAGCGCTAGAAAATGCAGTTGAAACTCTCTTCTCCAAAGATTTCCACCTTTTTCAAGCTATTAACGCAGCAGACTTGGGTTGTGCAACGGGTCCAAACACGTTCGCAGTGATTTCTACGATCAAGAGAATGATGGAAAAGAAATGCAGGGAATTGAATTGCCAAACACTGGAACTTCAGGTTTACATGAATGATCTTTTTGGAAACGATTTCAATACCCTCTTCAAAGGCCTGTCGTCTAAGGTTATTGGTAACAAATGCGAGGAAGTTTCTTGTTATGTGATGGGAGTACCAGGGTCTTTCCATGGCCGGCTTTTTCCTCGTAACAGCTTGCATTTAGTTCATTCGTCTTACAGTGTTCATTGGCTTACTCAGGCACCAAAAGGACTCACAAGTAGAGAAGGCTTGGCATTAAACAAGGGGAGGATTTACATATCAAAGACAAGCCCTCCTGTTGTAAGAGAAGCCTACTTATCTCAATTTCATGAAGATTTCACAATGTTTCTTAATGCTAGATCCCAAGAGGTGGTTCCAAATGGTTGTATGGTGTTGATACTTCGTGGTAGGCAATCTTCTGATCCTTCAGACATGCAGAGCTGCTTTATTTGGGAACTATTAGCTATAGCCATTGCTGAATTGGTTTCGCAGGGATTGATAGATGAAGATAAATTAGACACCTTCAATATACCTTGCTATTTTCCATCACTTGAGGAAGTGAAAGATATAGTGGAGAGGGACGGATCATTCACAATTGATCATATGGAGGGGTTTGAACTTGATAGCCTACAGATGCAAGAGAATGATAAATGGGTTAGAGGGGAAAAGTTTGCCAAGATTGTCAGGGCCTTCACAGAGCCTATAATTTCAAACCAGTTTGGACATGAAATCATGGACAAACTATATGACAAATTCACTCACATTGTAGTTTCAGATTTGGAAGCAAAGCTGCCGAAGACCACAAGTATCATCCTAGTGCTTTCCAAGATTGATGGATAG |
| >TcSd  ATGGAGCTAGCTACTAGGGGGAAGGTGAAAGAAGTGTTGTTCATGAACAGAGGAGAAGGAGAAAGTAGTTATGTACAAAACTCTTCTTTCACGGAAAAAGTGGCCTCAATGGCAATGCCAGCGCTAGAAAATGCAGTTGAAACTCTCTTCTCCAAAGATTTCCACCTTTTTCAAGCTATTAACGCAGCAGACTTGGGTTGTGCAACGGGTCCAAACACGTTCGCAGTGATTTCTACGATCAAGAGAATGATGGAAAGGAAATGCAGGGAATTGAATTGCCAAACACTGGAACTTCAGGTTTACATGAATGATCTTTTTGGAAACGATTTCAATACCCTCTTCAAAGGCCTGTCGTCTAAGGTTATTGGTAACAAATGCGAGGAAGTTTCTTGTTATGTGATGGGAGTACCAGGGTCTTTCCATGGCCGGCTTTTTCCTCGTAACAGCTTGCATTTAGTTCATTCGTCTTACAGTGTTCATTGGCTTACTCAGGCACCAAAAGGACTCACAAGCAGAGAAGGCTTGGCATTAAACAAGGGGAGGATTTACATATCAAAGACAAGCCCTCCTGTTGTAAGAGAAGCCTACTTATCTCAATTTCATGAAGATTTCACAATGTTTCTTAATGCTAGATCCCAAGAGGTGGTTCCAAATGGTTGTATGGTGTTGATACTTCGTGGTAGGCAATCTTCTGATCCTTCAGACATGCAGAGCTGCTTTATTTGGGAACTATTAGCTATAGCCATTGCTGAATTGGTTTCGCAGGGATTGATAGATGAAGATAAATTAGACACCTTCAATATACCTTGCTATTTTCCATCACTTGAGGAAGTGAAAGATATAGTGGAGAGGGACGGATCATTCACAATTGATCATATGGAGGGGTTTGAACTTGATAGCCTACAGATGCAAGAGAATGATAAATGGGTTAGAGGGGAAAAGTTTACCAAGATTGTCAGGGCCTTCACAGAGCCTATAATTTCAAACCAGTTTGGACATGAAATCATGGACAAACTATATGACAAATTCACTCACATTGTAGTTTCAGATTTGGAAGCAAAGCTGCCGAAGACCACAAGTATCATCCTAGTGCTTTCCAAGATTGTTGGATAG |
| >TcSe  ATGGAGCTAGCTACTAGGGGGAAGGTGAAAGAAGTGTTGTTCATGAACACAGGAGAAGGAGAAAGTAGTTATGTACAAAACTCTTCTTTCACAGAAAAAGTGGCCTCAATGGCAATGCCAGCGCTAGAAAATGCAGTTGAAACTCTCTTCTCCAAAGATTTCCACCTTTTTCAAGCTATTAACGCAGCAGACTTGGGTTGTGCAACGGGTCCAAACACGTTCGCAGTGATTTCTACGATCAAGAGAATGATGGAAAAGAAATGCAGGGAATTGAATTGCCAAACACTGGAACTTCAGGTTTACATGAATGATCTTTTTGGAAACGATTTCAATACCCTCTTCAAAGGCCTGTCGTCTAAGGTTATTGGTAACAAATGCGAGGAAGTTTCTTGTTATGTGATGGGAGTACCAGGGTCTTTCCATGGCCGGCTTTTTCCTCGTAACAGCTTGCATTTAGTTCATTCGTCTTACAGTGTTCATTGGCTTACTCAGGCACCAAAAGGACTCACAAGTAGAGAAGGCTTGGCATTAAACAAGGGGAGGATTTACATATCAAAGACAAGCCCTCCTGTTGTAAGAGAAGCCTACTTATCTCAATTTCATGAAGATTTCACAATGTTTCTCAACGCTAGATCCCAAGAGGTGGTTCCAAATGGTTGTATGGTGTTGATACTTCGTGGTAGGAAAGCTTCTGATCCTTCAGACATGGAGAGCTGCTTTACTTGGGAACTATTAGCTATAGCCATTGCTGAATTGGTTTCACAGGGATTGATAGATGAAGATAAATTAGACACCTTCAATATACCCTGCTATTTTCCATCACTTGAGGAAGTGAAAGATATAGTGGAGAGGGACGGATCATTCACAATTGATCATATGGAGGGGTTTGAACTTGATAGCCTACAGATGCAGGAGAATGATAAATGGGTTAGAGGGGAAAAGTTTGCCAAGATTGTCAGGGCCTTCACAGAGCCTATAATTTCAAACCAGTTTGGACATGAAATCATGGACAAACTATATGACAAATTCACTCACATTGTAGTTTCAGATTTGGAAGCAGAGCTACCGAAGACCACAAGTATCATCCTAGTGCTTTCCAAGATTGTTGGATAG |
| >TcSf  ATGGAGCTAGCTACTAGGGGGAAGGTGAAAGAAGTGTTGTTCATGAACAGAGGAGAAGGAGAAAGTAGTTATGTACAAAACTCTTCTTTCACGGAAAAAGTGGCCTCAATGGCAATGCCAGCGCTAGAAAATGCAGTTGAAACTCTCTTCTCCAAAGATTTCCACCTTTTTCAAGCTATTAACGCAGCAGACTTGGGTTGTGCAACGGGTCCAAACACGTTCGCAGTGATTTCTACGATCAAGAGAATGATGGAAAAGAAATGCAGGGAATTGAATTGCCAAACACTGGAACTTCAGGTTTACATGAATGATCTTTTTGGAAACGATTTCAATACCCTCTTCAAAGGCCTGTCGTCTAAGGTTATTGGTAACAAATGCGAGGAAGTTTCTTGTTATGTGATGGGAGTACCAGGGTCTTTCCATGGCCGGCTTTTTCCTCGTAACAGCTTGCATTTAGTTCATTCGTCTTACAGTGTTCATTGGCTTACTCAGGCACCAAAAGGACTCACAAGCAGAGAAGGCTTGGCATTAAACAAGGGGAGGATTTACATATCAAAGACAAGCCCTCCTGTTGTAAGAGAAGCCTACTTATCTCAATTTCATGAAGATTTCACAATGTTTCTTAATGCTAGATCCCAAGAGGTGGTTCCAAATGGTTGTATGGTGTTGATACTTCGTGGTAGGCAATCTTCTGATCCTTCAGACATGCAGAGCTGCTTTATTTGGGAACTATTAGCTATAGCCATTGCTGAATTGGTTTCGCAGGGATTGATAGATGAAGATAAATTAGACACCTTCAATATACCTTGCTATTTTCCATCACTTGAGGAAGTGAAAGATATAGTGGAGAGGGACGGATCATTCACAATTGATCATATGGAGGGGTTTGAACTTGATAGCCTACAGATGCAAGAGAATGATAAATGGGTTAGAGGGGAAAAGTTTACCAAGATTGTCAGGGCCTTCACAGAGCCTATAATTTCAAACCAGTTTGGACATGAAATCATGGACAAACTATATGACAAATTCACTCACATTGTAGTTTCAGATTTGGAAGCAAAGCTGCCGAAGACCACAAGTATCATCCTAGTGCTTTCCAAGATTGTTGGATAG |
| >TcSg  ATGGAGCTAGCTACTAGGGGGAAGGTGAAAGAAGTGTTGTTCATGAACAGAGGAGAAGGAGAAAGTAGTTATGTACAAAACTCTTCTTTCACGGAAAAAGTGGCCTCAATGGCAATGCCAGCGCTAGAAAATGCAGTTGAAACTCTCTTCTCCAAAGATTTCCACCTTTTTCAAGCTATTAACGCAGCAGACTTGGGTTGTGCAACGGGTCCAAACACGTTCGCAGTGATTTCTACGATCAAGAGAATGATGGAAAAGAAATGCAGGGAATTGAATTGCCAAACACTGGAACTTCAGGTTTACATGAATGATCTTTTTGGAAACGATTTCAATACCCTCTTCAAAGGCCTGTCGTCTAAGGTTATTGGTAACAAATGCGAGGAAGTTTCTTGTTATGTGATGGGAGTACCAGGGTCTTTCCATGGCCGGCTTTTTCCTCGTAACAGCTTACATTTAGTTTATTCCTCTTACAGTGTTCATTGGCTTACTCAGGCACCAAAAGGACTCACAAGCAGAGAAGGCTTGGCATTAAACAAGGGGAAGATTTACATATCAAAGACAAGCCCTCCTGTTGTAAGAGAAGCCTACTTATCTCAATTTCATGAAGATTTCACAATGTTTCTCAATGCTAGATCCCAAGAGATGGTTCCAAATGGTTGTATGGTGTTGATACTTCGTGGTAGGCAATGTTTTGATCCTTCAGACATGCAGAGCTGCTTTACTTGGGAACTATTAGCTTTAGCCATTGCTGAATTGGTTTCACAGGGATTGATAGATGAAGATAAATTAGACACCTTCAATATACCCAGCTATTTTGCATCACTTGAGGAAGTGAAAGATATAGTGGAGAGGGACGGATCATTCACAATTGATCATATAGAGGGGTTTGATCTTGATAGCGTAGAAATGCAGGAGAATGATAAATGGGTTAGAGGGGAAAAGTTTACCAAGGTTGTCAGGGCCTTCTCAGAGCCTATAATTTCAAGCCAGTTTGGACATGAAATCATGGACAAACTATATGACAAATTCACTCACATTGTAGTTTCAGATTTGGAAGCAAAGCTACCGAAGACCACAAGTATCATCCTAGTGCTTTCCAAGATTGTTGGATAG |

**Table S6.** Coding region nucleotide (upper portion) and amino acid (bottom portion) sequence comparison of TcSs

|  | TcSa | TcSb | TcSc | TcSd | TcSe | TcSf | TcSg |
| --- | --- | --- | --- | --- | --- | --- | --- |
| TcSa | - | 99.82 | 99.91 | 99.55 | 99.01 | 99.64 | 97.48 |
| TcSb | 99.73 | - | 99.73 | 98.92 | 98.38 | 99.19 | 94.86 |
| TcSc | 99.73 | 99.46 | - | 98.92 | 98.38 | 99.19 | 94.86 |
| TcSd | 99.19 | 99.37 | 99.46 | - | 98.56 | 99.91 | 97.75 |
| TcSe | 98.65 | 98.83 | 98.92 | 97.84 | - | 98.65 | 97.57 |
| TcSf | 99.46 | 99.46 | 99.55 | 99.73 | 98.11 | - | 97.84 |
| TcSg | 95.14 | 97.30 | 97.39 | 95.41 | 94.59 | 95.68 | - |

**Table S7.** Promoter sequences of *TcSs* in different tea resources

| >TcSP1  GTAGGTCAAGAATGTGGGTTTATATGCATTATTTTGAGGGCGTTACAGCTTTGCTGTCGTTTTTGGGCTGATTTGCTGTTATTTTTGGGCTGATTTATCATATGTTTAAGCTTCATTTTGAACTTAAAAAAAAAAAAAAAATAGAGCATCAAATAATCAATTAATCTAAGATGATTTAATGAACTTATATCAGGCAAGATTGAATTTCATTTTGATCTTCAAAAATAACATTATGATAGGATAAATTAATATGAATAATTTGAGATATGTCAATCAAATTAATTCTTTCAAATAATCAAAAACATAATCAAAACATCAAAATCAAATATGTCTAACAAAATAAATAAGAATACGAAAAGTGTACTGAAGTTTTGTATTTTTTGTATTTATATTTTTGGTAATTAGTGAATTTCTTAAAGATTTTTTTTACAAATATTATACTTTTTAAGTATATTTATGTTAGGTGTATCTAAAATTTTTAAAAATTGCTTAGAATATTAAAAAAAATTTAGTGAAAACAAAAAAAAAAAAATATGAAAAATAAAAAATTTCAATTTTCTAAAAAGTGAATCCTGAAAATTCAAACCAAATAAACTAAATTTGTTGTTAGAGTATTAAATAATTTCTCTATAACCGTTTGTCAATTTTAATACTTCTTTTAAAGTCTTAAGATTGATATTTAAACTCATTAAATTGGATTAGAATTATTTTTTAAAGTTTTTCTTAGATGGGTTGGATTTGGAAATGTTTCTTCCAGCTTGCCCTAGCATGATTATTAATATATATTATGTCCATGTAAAATTTTACTATAAATACTTCTTATCGTATGTATTCTTAACTTATATTATACTTTTAAGTTATACAAATTTCTTTTTCAGTTATGATTAATTTTTTTCTTTAGTTGCGATCTTATTTTAGTTATGACTCATAAGATCTTTGGTTATATCTCATGTATTATTTGGTTATGACTTTTTTTTTTAGTTATGACTTTAAAGTATGAAATTATGTCATTTTTTTTTTAGTTATAAGGTACATACGATACATACCCTTTAAATTTTTCTCGTATGTACATATGAACATATTAATATCTGTGTATATATAATATACTAAATTTTACTTTTTTAAAATAAAAAGATATACTTAAAATGAAAAATGTATTTATTTAAGTTAATATTTAGGCCAAGTTTGGCATGTTCAGGAAAAGCTAAAAAGTTAAAAAGTTACTCAAAAAGTTAAAAAGTTAACTCTATTTACCAAAAGTCAACAATTACGGCTGCACAACTCCCAATCCTAATTTTCTTCTACAAACCAATTTTTATAATTTTTTATTACACATTTTTTCATAAAAAGTCAAAAAGTTAACCCAAACATCTATATTTTCTTCCTCAATTTTTTAGTCCAAAAAGATAAAAAGTACACTTATTTAGGACACTCCAAACAAACCCTTAAGATGTAATGACTAAATATTAAGGTGTAATTTTAATTAGCTTGATTTTGATTTTAAATTTTTAACTTTGCCTCAATTTGGTCAAATGGATTACAACTCTTAATATTTTTTAATAAAAAATATGTACATAAGTGTCATGTGTCTCAAAATTATGAATTTATCTAACTCAGTAAGTGAATCTCAACTAATTTAGTGATAATTTTTCTTAAAAAACACTAATGATATTATTAATTTGAACAAATTGACGTAAAATCTAAAATTAAGGATCAAAATCAAATCAATTAAAAATGCAAAAACCAAAATAAAACAAAAGATAAAATATAAGGACATCGGCGTAATTTACCCACAAAATTATCATTTTTCAGTTTTATAATATTTAAAATTGTTTATATGAGTTTGTTGGGCATGTTCGAGATTGTACTAGCAAGATTTTAAGGCTAGCTTTGGTAGAGATTTTGGGTTTGTTTGATTTGTATCTTATGATATAATTTTTAATTTTTTTTTTAAATTTTGTTTGGTTTAAATTTTTTGATTTTTTTTTTTCAAAATCATTTTTTTTTTCGTATATATCAATCAGTCACTTTTTCTCTCTTCTCTTTCCTTCTCAATCATTTTTTTTTTCTCACACACATCTACTCAAACTACAATAAAATATCAAATCATCCCAAACTCCCAAACTATTTTCAAAATTTACAACCAAACAAACCAAAAAAAATTCTAAACTCTCTCTGTAAAAAAAAAAATTTAAAAATTTATCTCAAAATAAAAACCAAACACACCCTTTTAGTTTCAAAAACTGAAAAAAATATTTGGTGTTATTGGACGTCACGTGGCGTACTACTTAGAAAAATATTTGAGGACCCGAAATTGTTTGGCTCGAAATTGGCTCGAAACTGAAAACGACGTCGTTTTGGATTCTTTTAGCGGGTAACATGCAATAAATTTTTTTTATCGGTAAGATATATTACAATTCTCTTATCGGTAAGATAATTCAAAACGACGTCGTTTTGGATTCGGGACAAATTCGAACTTGGAATGTTCGGACCCTGTAGCATCGTTGGTACTACTTACCAATAATAATATATCATGTTTCTATAATTTTTTAATCACTTAATATAAAATTATAAGTCTCATTTTTTTTTATTAATTAAAATACTTTTGTGTATCACGTGCAAAATCAACCAATAATTTCTCAAAAACAATTCCTAACTTAGGCGTAGCCGAACACCCAGACTATAGATAGGCTTTCAGGCCATTATTCACATCACTACTGTGGTAGCTGGCCTCTTTGCTATAAAAATTAGTGCTCTTCTGGTTATTCATATTCATATCACCGCTGTGGCAGCTGGCCTCTTTGATATAAAAATTACTTTTCTGACGAGGC |
| --- |
| >TcSP2  GTAGGTCAAGAATGTGGGTTTATATGCATTATTTTGAGGGCGTTACAGCTTTGCTGTCGTTTTTGGGCTGATTTGCTGTTATTTTTGGGCTGATTTATCATATGTTTAAGCTTCATTTTGAACTTAAAAAAAAAAAAAAAAAAAAAATTAGAGCATCAAATAATCAATTAATCTAAGATGATTTAAGGAACTTATACCAGGCAAGATTGAATTTCATTTTGATCTTCAAAAATAACATTATGATAGGATAAATTAATATGAATAATTTGAGATATGTCAATCAAATTAATTCTTTCAAATAATCAAAAACATAATCAAAACATCAAAATCAAATATGTCTAACAAAATAAATAAGAATACGAAAAGTGTACTGAAGTTTTGTATTTTTTGTATTTATATTTTTGGTAATTAGTGAATTTCTTAAAGATTTTTTTTTACAAATATTATACTTTTTAAGTATATTTATGTTAGGTGTATCTAAAATTTTTTAAAATTGCTTAGAATATTAAAAAAAATTTAGTGAAAACAAAAAAAAAAAAATGAAAAATAAAAAATTTCAATTTTCTAAAAAGTGAATCCTGAAAATTCAAACCAAACAAACTAAATTTGTTGTTAGAGTATTAAATAATTTCTCTATAACCGTTTGTCAATTTTAATACTTCTTTTAAAGTCTTAAGATTGATATTTAAACTCATTAAATTGGATTAGAATTATTTTTTAAAGTTTTTCTTAGATGGGTTGGATTTGGAAATGTTTCTTCCTGCTTGCCCTAGCATGATTATTAATATATATTATGTCCATGTAAAATTTTACTATAAATACTTTTTATCGTATGTATTCTTACCTTATATAATACTTTTAAGTTATATAAATTTCTTTTTCAGTTATGATTAATTTTTTTCTTTAGTTGCGATCTTATTTTAGTTATGACTCATAAGATCTTTGGTTATATCTCATGTATTATTTGGTTATGACTTTTTTTTTTAGTTATGACTTTAAAGTATGAAATTATGTCATTTTTTTTTTAGTTATAAGGTACATACGATACATACCCTTTAAATTTTTCTCGTATGTACATATGAACATATTAATATCTGTGTATATATAATTTACTAAATTTTACTTTTTTAAAATAAAAAGATATACTTAAAATGAAAAATGTATTTATTTAAGTTAATATTTAAGATGTAATGACTAAATATTAAGGTGTAATTTTAATTAGCTTGATTTTGATTTTAAATTTTTAACTTTGCCTCAATTTGGTCAAATGGATTACAACTCTTAATATTTTTTAATAAAAAATATGTACATAAGTGTCATGTGTCTCAAAATTATGAATTTATCTAACTCAGTAAGTGAACTCAACTAATTTAGTGATAATTTTTCTTAAAAAACACTAATGATATTATTAATTTGAACAAATTGACGTAAAATCTAAAATTAAGGATCAAAATCAAATCAATTAAAAATGCAAAAACCAAAATAAAACAAAAGATAAAATATAAGGACATCGGCGTAATTTACCCACAAAATTATCATTTTTCAGTTTTATAATATTTAAAATTGTTTATATGAGTTTGTTGGGCATGTTCGAGATTGTCCTAGCAAGATTTTAAGGCTAGCTTTGGTAGAGATTTTGGGTTTGTTTGATTTGTATCTCATGATATAATTTTTAATTTTTTTTTAAATTTTGTTTGGTTTAAATTTTTTGATTTTTTTTTTCAAAATCACTTTTTTTTTTCGTATATATCAATCAGTCACTTTTTCTCTCTTCTCTTTCCTTCTCAATCATTTTTTTTTCTCACACACATCTACTCAAACTACAATAAAATATCAAATCATCCCAAACTCCCAAACTATTTTCAAAATTTACAACCAAACAAACCAAAAAAAATTCTAAACTCTCTCTGTAAAAAAAAATTAAAAAATTTATCTCAAAATAAAAACCAAACACACCCTTTTAGTTTCAGAAACTGAAAAAAATATTTGGTGTTATTGGACGTCACGTGGCGTACTACTTAGAAAAATATTTGAGGACCCGAAATTGTTTGGCTCGAAATTGGCTCGAAACTGAAAACGACGTCGTTTTGGATTCTTTTAGCGAGTAACATGTAATAAAATTTTTTTATCGGTAAGATATATTACAATTCTCTTATCGGTAAGATAATTCAAAACGACGTCGTTTTGGATTCGGGACAAATTCGAACTTGAAATGTTCGGGTCCTGTAGCAGCGTTGGTACTACTTACCAATAATAATATATCATGTTTCTATAATTTTTTAATCACTTAGGCCTCGTTTGGCAAGAGGCTATTTTAGTCAATTAGTCAAAAAGTTACTAAATTAGCCAAAAAGCCAACAACCCATACCAAAAAGTCAAAGATTCTCTTTTCTCTCTACTTTTTGCACATCTTTTCGTAATTAGCCAAAAATTCATCCCAAACATACAAATTTTTTTTTACCTACCTTTTTAACCAAATAGTCAAAAAGTTACTAAATTAGCCTGTTCCTAAACGAGGCCTTAATATAAAATTATAAGTCTCATTTTTTTTTATTAATTAAAATACTTTTGTGTATCACGTGCAAAATCAACCAATAATTTCTCAAAAACAATTCCTAACTTAGGCGTAGCCGAACACCCAGACTATAGATAGGCTTTCAGGCCATTATTCACATCACTACTGTGGTAGCTGGCCTCTTTGCTATAAAAATTAGTGCTTTTCTGGTTATTCATATTCATATCACTGCTGTGGCAGCTGGCCTCTTTGATATAAAAATTACTTTTCTGACGAGGC |
| >TcSP3  AGGTCAAGAATGTGGGTTTATATGCATTATTTTGAAGGCGTTACAGTTTTGCTGTCATTTTTGGGCTGATTTGCTGTTATTTTTGGGCTGATTTATCATATGTTTAAGCTTCATTTTGAACTTAAAAAAAAAAAAAAAATTAGAGCATCAAATAATCAATTAATCTAAGATGATTTAAGGAACTTATACCAGGCAAGATTGAATTTCATTTTGATCTTCAAAAATAACATTATGATAGGATAAATTAATATGAATAATTTGAGATATGTCAATCAAATTAATTCTTTCAAATAATCAAAAACATAATCAAAACATCAAAATCAAATATGTCTAACAAAATAAATAAGAATACGAAAACTGTACTGAAGTTTTGTATTTTTTGTATTTATATTTTTGGTAATTAGTGAATTTCTTAAAGATTTTTTTTTTACAAATATTATACTTTTTAAGTATATTTATGTTAGGTGTATCTAAAATTTTTTAAAATTGCTTAGAATATTAAAAAAAATTTAGTGAAAACAAAAAAAAAAAATGAAAAATAAAAAATTTCAATTTTCTAAAAAGTGAATCCTGAAAATTCAAACCAAACAAACTAAATTTGTTGTTAGAGTATTAAATAATTTCTCTATAACCGTTTGTCAATTTTAATACTTCTTTTAAAGTCTTAAGATTGATATTTAAACTCATTAAATTGGATTAGAATTATTTTTTAAAGTTTTTCTTAGATGGGTTGGATTTGGAAATGTTTCTTCCTGCTTGCCCTAGCATGATTATTAATATATATTATGTCCATGTAAAATTTTACTATAAATACTTTTTATCGTATGTATTCTTACCTTATATTATACTTTTAAGTTATACAAATTTCTTTTTCAGTTATGATTAATTTTTTTCTTTAGTTGCGATCTTATTTTAGTTATGACTCATAAGATCTTTGGTTATATCTCATGTATTATTTGGTTATGACTTTTTTTTTTAGTTATGACTTTAAAGTATGAAATTATGTCATTTTTTTTTTAGTTATAAGGTACATACGATACATACCCTTTAAATTTTTCTCGTATGTACATATGAACATATTAATATCTGTGTATATATAATTTACTAAATTTTACTTTTTTAAAATAAAAAGATATACTTAAAATGAAAAATGTATTTATTTAAGTTAATATTTAAGATGTAATGACTAAATATTAAGGTGTAATTTTAATTAGCTTGATTTTGATTTTAAATTTTTAACTTTGCCTCAATTTGGTCAAATGGATTACAACTCTTAATATTTTTTAATAAAAAATATGTACATAAGTGTCATGTGTCTCAAAATTATGAATTTATCTAACTCAGTAAGTGAATCTCAACTAATTTAGTGATAATTTTTCTTAAAAAACACTAATGATATTATTAATTTGAACAAATTGACGTAAAATCTAAAATTAAGGATCAAAATCAAATCAATTAAAAATGCAAAAACCAAAATAAAACAAAAGATAAAATATAAGGACATCGGCGTAATTTACCCACAAAATTATCATTTTTCAGTTTTATAATATTTAAAATTGTTTATATGAGTTTGTTGGGCATGTTCGAGATTGTCCTAGCAAGATTTTAAGGCTAGCTTTGGTAGAGATTTTGGGTTTGTTTGGTTTAAATTTTTTGATTTTTTTTTCAAAATCACTTTTTTTTTCGTATATATCAATCAGTCACTTTTTCTCTCTTCTCTTTCCTTCTCAATCATTTTTTTTTTCTCACACACATCTACTCAAACTACAATAAAATATCAAATCATCCCAAACTCCCAGACTATTTTCAAAATTTACAACCAAACAAACCAAAAAAAATTCTAAACTCTCTCTGTAAAAAAAAATTAAAAAATTTATCTCAAAATAAAAACCAAACACACCCTTTTAGTTTCAAAAACTGAAAAAAATATTTGGTGTTATTGGACGTCACGTGGCGTACTACTTAGAAAAATATTTGAGGACCCGAAATTGtTTGGCTCGAAATTGGCTCGAAACTGAAAACGACGTCGTTTTGGATTCTTTTAGCGAGTAACATGTAATAAAATTTTTTTATCGGTAAGATATATTACAATTCTCTTATCGGTAAGATAATTCAAAATGACGTCGTTTtGGATTCGGGACAAATTCGAACTTGAAATGTTCGGGTCCTGTAGCAGCGTTGGTACTACTTACCAATAATAATATATCATGTTTCTATAATTTTTTAATCACTTAGGCCTCGTTTGGCAAGAGGCTATTTTAGTCAATTAGTCAAAAAGTTACTAAATTAGCCAAAAAGCCAACAACCCATACCAAAAAGTCAAAGATTCTCTTTTCTCTCTACTTTTTGCACATCTTTTCGTAATTAGCCAAAAATTCATCCCAAACATACAAATTTTTTTTACCTACCTTTTTAACCAAATAGTCAAAAAGTTACTAAATTAGCCTGTTCCCAAACGAGGCCTTAATATAAAATTATAAGTCTCATTTTTTTTTATTAATTAAAATACTTTTGTGTATCACGTGCAAAATCAACCAATAATTTCTCAAAAACAATTCCTAACTTAGGCGTAGCCGAACACCCAGACTATAGATAGGCTTTCAGGCCATTATTCACATCACTACTGTGGTAGCTGGCCTCTTTGCTATAAAAATTAGTGCTTTTCTGGTTATTCATATTCATATCGCTGCTGTGGCAGCTGGCCTCTTTGATATAAAAATTACTTTTCTGACGAGGC |
| >TcSP4  GTAGGTCAAGAATGTGGGTTTATATGCATTATTTTGAGGGCGTTACAGCTTTGCTGTCGTTTTTGGGCTGATTTGCTGTTATTTTTGGGCTGATTTATCATATGTTTAAGCTTCATTTTGAACTTAAAAAAAAAAAAAAAAAATTAGAGCATCAAATAATCAATTAATCTAAGATGATTTAAGGAACTTATACCAGGCAAGATTGAATTTCATTTTGATCTTCAAAAATAACATTATGATAGGATAAATTAATATGAATAATTTGAGATATGTCAATCAAATTAATTCTTTCAAATAATCAAAAACATAATCAAAACATCAAAATCAAATATGTCTAACAAAATAAATAAGAATACGAAAAGTGTACTGAAGTTTTGTATTTTTTGTATTTATATTTTTGGTAATTAGTGAATTTCTTAAAGATTTTTTTTTACAAATATTATACTTTTTAAGTATATTTATGTTAGGTGTATCTAAAATTTTTTAAAATTGCTTAGAATATTAAAAAAAATTTAGTGAAAACAAAAAAAAAAAAAATGAAAAATAAAAAATTTCAATTTTCTAAAAAGTGAATCCTGAAAATTCAAACCAAACAAACTAAATTTGTTGTTAGAGTATTAAATAATTTCTCTATAACCGTTTGTCAATTTTAATACTTCTTTTAAAGTCTTAAGATTGATATTTAAACTCATTAAATTGGATTAGAATTATTTTTTAAAGTTTTTCTTAGATGGGTTGGATTTGGAAATGTTTCTTCCTGCTTGCCCTAGCATGATTATTAATATATATTATGTCCATGTAAAATTTTACTATAAATACTTTTTATCGTATGTATTCTTACCTTATATAATACTTTTAAGTTATACAAATTTCTTTTTCAGTTATGATTAATTTTTTTCTTTAGTTGCGATCTTATTTTAGTTATGATTCATAAGATCTTTGGTTATATCTCATGTATTATTTGGTTATGACTTTTTTTTTTTAGTTATGACTTTAAAGTATGAAATTATGTCATTTTTTTTTTAGTTATAAGGTACATACGATACATACCCTTTAAATTTTTCTCGTATGTACATATGAACATATTAATATCTGTGTATATATAATTTACTAAATTTTACTTTTTAAAATAAAAAGATATACTTAAAATGAAAAATGTATTTATTTAAGTTAATATTTAAGATGTAATGACTAAATATTAAGGTGTAATTTTAATTAGCTTGATTTTGATTTTAAATTTTTAACTTTGCCTTAATTTAGTCAAATGGATTACAACTCTTAATATTTTTTAATAAAAAATATGTACATAAGTGTCATGTGTCTCAAAATTATGAATTTATCTAACTCAGTAAGTGAATCTCAACTAATTTAGTGATAATTTTTCTTAAAAAACACTAATGATATTATTAATTTGAACAAATTGACGTAAAATCTAAAATTAATGATCAAAATCAAATCAATTAAAAATGCAAAAACCAAAATAAAACAAAAGATAAAATATAAGGACATCGGCGTAATTTACCCACAAAATTATCATTTTTCAGTTTTATAATATTTAAAATTGTTTATATGAGTTTGTTGGGCATGTTCGAGATTGTCCTAGCAAGATTTTAAGGCTAGCTTTGGTAGAGATTTTGGGTTTGTTTGATTTGTATCTCATGATATAATTTTTAATTTTTTTTTTAAATTTTGTTTGGTTTAAATTTTTTGATTTTTTTTTTTCAAAATCACTTTTTTTTTTCGTATATATCAATCAGTCACTTTTTCTCTCTTCTCTTTCCTTCTCAATCATTTTTTTTTTCTCACACACATCTACTCAAACTACAATAAAATATCAAATCATCCCAAACTCCCAAACTATTTTCAAAATTTACAACCAAACAAACCAAAAAAAATTCTAAACTCTCTCTGCAAAAAAAAATTAAAAAATTTATCTCAAAATAAAAACCAAACACACCCTTTTAGTTTCAAAAACTGAAAAAAATATTTGGTGTTATTGGACGTCACGTGGCGTACTACTTAGAAAAATATTTGAGGACCCGAAATTGTTTGGCTCGAAATTGGCTCGAAACTGAAAACGACGTCGTTTTGGATTCTTTTAGCGAGTAACATAAAATAAAATTTTTTTATCGGTAAGATATATTACAATTCTCTTATCGGTAAGATAATTCAAAACGACGTCGTTTTGGATTCGGGACAAATTCGAACTTGAAATGTTCGGGTCCTGTAGCAGCGTTGGTACTACTTACCAATAATAATATATCATGTTTCTATAATTTTTTAATCACTTAGGCCTCGTTTGGCAAGAGGCTATTTTAGTCAATTAGTCAAAAAGTTACTAAATTAGCCAAAAAGCCAACAACCCATACCAAAAAGTCAAAGATTCTCTTTTCTCTCTACTTTTTGCACATCTTTTCGTAATTAGTCAAAAATTCATCCCAAACATACAAATTTTTTTTACCTACCTTTTTAAGCAAATAGTCAAAAAGTTACTAAATTAGCCTGTTCCCAAACGAGGCCTTAATATAAAATTATAAGTCTCATTTTTTTTATTAATTAAAATACTTTTGTGTATCACGTGCAAAATCAACCAATAATTTCTCAAAAACAATTCCTAACTTAGGCGTAGCCGAACACCCAGACTATAGATAGGCTTTCAGGCCATTATTCACATCACTACTGTGGTAGCTGGCCTCTTTGCTATAAAAATTAGTGCTTTTCTGGTTATTCATATTCATATCACTGCTGTGGCAGCTGGCCTCTTTGATATAAAAATTACTTTTCTGACGAGGC |
| >TcSP5  GTAGGTCAAGAATGTGGGTTTATATGCATTATTTTGAGGGCGTTACAGCTTTGCTGTCGTTTTTGGGCTGATTTGCTGTTATTTTTGGGCTGATTTATCATATGTTTAAGCTTCATTTTGAACTTAAAAAACAAAAAAAAAAAAATTAGAGCATCAAATAATCAATTAATCTAAGATGATTTAAGGAACTTATACCAGGCAAGATTGAATTTCATTTTGATCTTCAAAAATAACATTATGATAGGATAAATTAATATGAATAATTTGAGATATGTCAATCAAATTAATTCTTTCAAATAATCAAAAACATAATCAAAACATCAAAATCAAATATGTCTAACAAAATAAATAAGAATACGAAAAGTGTACTGAAGTTTTGTAATTTTTGTATTTATATTTTTGGTAATTAGTGAATTTCTTAAAGATTTTTTTTTACAAATATTATACTTTTTAAGTATATTTATGTTAGGTGTATCTAAAATTTTTTAAAATTGCTTAGAATATTAAAAAAAATTTAGTGAAAACAAAAAAAAAAAAAATGAAAAATAAAAAATTTCGATTTTCTAAAAAGTGAATCCTGAAAATTCAAACCAAACAAACTAAATTTGTTGTTAGAGTATTAAATAATTTCTCTATAACCGTTTGTCAATTTTAATACTTCTTTTAAAGTCTTAAGATTGATATTTAAACTCATTAAATTGGATTAGAATTATTTTTTAAAGTTTTTCTTAGATGGGTTGGATTTGGAAATGTTTCTTCCTGCTTGCCCTAGCATGATTATTAATATATATTATGTCCATGTAAAATTTTACTATAAATACTTTTTATCGTATGTATTCTTACCTTATATAATACTTTTAAGTTATATAAATTTCTTTTTCAGTTATGATTAATTTTTTTCTTTAGTTGCGATCTTATTTTAGTTATGACTCATAAGATCTTTGGTTATATCTCATGTATTATTTGGTTATGACTTTTTTTTTTTAGTTATGACTTTAAAGTATGAAATTATGTCATTTTTTTTTTAGTTATAAGGTACATACGATACATACCCTTTAAATTTTTCTCGTATGTACATATGAACATATTAATATCTGTGTATATATAATTTACTAAATTTTACTTTTTTAAAATAAAAAGATATACTTAAAATGAAAAATGTATTTATTTAAGTTAATATTTAAGATGTAATGACTAAATATTAAGGTGTAATTTTAATTAGCTTGATTTTGATTTTAAATTTTTAACTTTGCCTCAATTTGGTCAAATGGATTACAACTCTTAATATTTTTTAATAAAAAATATGTACATAAGTGTCATGTGTCTCAAAATTATGAATTTATCTAACTCAGTAAGTGAACTCAACTAATTTAGTGATAATTTTTCTTAAAAAACACTAATGATATTATTAATTTGAACAAATTGACGTAAAATCTAAAATTAAGGATCAAAATCAAATCAATTGAAAATGCAAAAACCAAAATAAAACAAAAGATAAAATATAAGGACATCGGCGTAATTTACCCACAAAATTATCATTTTTCAGTTTTATAATATTTAAAATTGTTTATATGAGTTTGTTGGGCATGTTCGAGATTGTCCTAGCAAGATTTTAAGGCTAGCTTTGGTAGAGATTTTGGGTTTGTTTGATTTGTATCTCATGATATAATTTTTAATTTTTTTTTTAAATTTTGTTTGGTTTAAATTTTTTGATTTTTTTTTTCAAAATCACTTTTTTTTTCGTATATATCAATCAGTCACTTTTTCTCTCTTCTCTTTCCTTCTCAATCATTTTTTTTTTCTCACACACATCTACTCAAACTACAATAAAATATCAAATCATCCCAAACTCCCAAACTATTTTCAAAATTTACAACCAAACAAACCAAAAAAAATTCTAAACTCTCTCTGTAAAAAAAAATTAAAAAATTTATCTCAAAATAAAAACCAAACACACCCTTTTAGTTTCAGAAACTGAAAAAAATATTTGGTGTTATTGGACGTCACGTGGCGTACTACTTAGAAAAATATTTGAGGACCCGAAATTGTTTGGCTCGAAATTGGCTCGAAACTGAAAACGACGGCGTTTTGGATTCTTTTAGCGAGTAACATGTAATAAAATTTTTTTATCGGTAAGATATATTACAATTCTCTTATCGGTAAGATAATTCAAAACGACGTCGTTTTGGATTCGGGACAAATTCGAACTTGAAATGTTCGGGTCCTGTAGCAGCGTTGGTACTACTTACCAATAATAATATATCATGTTTCTATAATTTTTTAATCACTTAGGCCTCGTTTGGCAAGAGGCTATTTTAGTCAATTAGTCAAAAAGTTACTAAATTAGCCAAAAAACCAACAACCCATACCAAAAAGTCAAAGATTCTCTTTTCTCTCTACTTTTTGCACATCTTTTCGTAATTAGCCAAAAATTCATCCCAAACATACAAATTTTTTTTACCTACCTTTTTAACCAAATAGTCAAAAAGTTACTAAATTAGCCTGTTCCTAAACGAGGCCTTAATATAAAATTATAAGTCTCATTTTTTTTTATTAATTAAAATACTTTTGTGTATCACGTGCAAAATCAACCAATAATTTCTCAAAAACAATTCCTAACTTAGGCGTAGCCGAACACCCAGACTATAGATAGGCTTTCAGGCCATTATTCACATCACTACTGTGGTAGCTGGCCTCTTTGCTATAAAAATTAGTGCTTTTCTGGTTATTCATATTCATATCACTGCTGTGGCAGCTGGCCTCTTTGATATAAAAATTACTTTTCTGACGAGGC |

**Table S8.** Comparision of *TcS* promoter sequences in different tea resources

|  | TcSP1 | TcSP2 | TcSP3 | TcSP4 | TcSP5 |
| --- | --- | --- | --- | --- | --- |
| TcSP1 | - | - | - | - | - |
| TcSP2 | 82.28 | - | - | - | - |
| TcSP3 | 80.70 | 97.35 | - | - | - |
| TcSP4 | 82.32 | 99.11 | 97.17 | - | - |
| TcSP5 | 82.32 | 99.50 | 97.25 | 99.14 | - |

**Table S9.** Phenotypic variations of caffeine and theacrine co-explained by *TcS* genotype of “ZC × RY” F_1_ population

| Season | caffeine | |  | theacrine | |
| --- | --- | --- | --- | --- | --- |
|  | *P* value | *R*^2^ (%) |  | *P* value | *R*^2^ (%) |
| Spring | 3.15E-107 | 95.53 |  | 1.30E-129 | 97.69 |
| Autumn | 3.50E-110 | 97.16 |  | 2.50E-131 | 98.58 |

*P*, significant level for association; *R^2^*, percentage of the phenotypic variance explained.
